# The Effectiveness of Self-Management Mobile Phone and Tablet Apps in Long-term Condition Management: A Systematic Review

**DOI:** 10.2196/jmir.4883

**Published:** 2016-05-16

**Authors:** Lisa Whitehead, Philippa Seaton

**Affiliations:** ^1^ School of Nursing and Midwifery Edith Cowan University Joondalup Australia; ^2^ Centre of Postgraduate Nursing Studies University of Otago Christchurch New Zealand

**Keywords:** mobile phone, apps, telemedicine, mHealth, self-management, chronic disease, diabetes mellitus, cardiovascular diseases, lung diseases, systematic review

## Abstract

**Background:**

Long-term conditions and their concomitant management place considerable pressure on patients, communities, and health care systems worldwide. International clinical guidelines on the majority of long-term conditions recommend the inclusion of self-management programs in routine management. Self-management programs have been associated with improved health outcomes; however, the successful and sustainable transfer of research programs into clinical practice has been inconsistent. Recent developments in mobile technology, such as mobile phone and tablet computer apps, could help in developing a platform for the delivery of self-management interventions that are adaptable, of low cost, and easily accessible.

**Objective:**

We conducted a systematic review to assess the effectiveness of mobile phone and tablet apps in self-management of key symptoms of long-term conditions.

**Methods:**

We searched PubMed, Embase, EBSCO databases, the Cochrane Library, and The Joanna Briggs Institute Library for randomized controlled trials that assessed the effectiveness of mobile phone and tablet apps in self-management of diabetes mellitus, cardiovascular disease, and chronic lung diseases from 2005–2016. We searched registers of current and ongoing trials, as well as the gray literature. We then checked the reference lists of all primary studies and review papers for additional references. The last search was run in February 2016.

**Results:**

Of the 9 papers we reviewed, 6 of the interventions demonstrated a statistically significant improvement in the primary measure of clinical outcome. Where the intervention comprised an app only, 3 studies demonstrated a statistically significant improvement. Interventions to address diabetes mellitus (5/9) were the most common, followed by chronic lung disease (3/9) and cardiovascular disease (1/9). A total of 3 studies included multiple intervention groups using permutations of an intervention involving an app. The duration of the intervention ranged from 6 weeks to 1 year, and final follow-up data ranged from 3 months to 1 year. Sample size ranged from 48 to 288 participants.

**Conclusions:**

The evidence indicates the potential of apps in improving symptom management through self-management interventions. The use of apps in mHealth has the potential to improve health outcomes among those living with chronic diseases through enhanced symptom control. Further innovation, optimization, and rigorous research around the potential of apps in mHealth technology will move the field toward the reality of improved health care delivery and outcomes.

## Introduction

The number of people living with 1 or more chronic disease continues to increase worldwide [1]. Improvement in living conditions and treatment has increased the life expectancy of people with chronic conditions; however, without effective management, quality of life may be poor. Empowering and engaging people with chronic diseases to manage their own health is vital. Several barriers have been identified in the ability of the individual and health care providers to work together to promote self-management [2]. Having access to timely information, assessment, and treatment are all vital in the management of long-term conditions [3]. mHealth interventions offer the potential to overcome many of the traditional barriers by offering convenience and care in a natural environment and minimizing the barriers of distance, time, and cost. For the clinician, mHealth interventions offer the ability to evaluate a prescribed course of action, monitor adverse events, and identify areas for improvement [4,5].

For the past decade, mHealth has been constantly expanding as a subdivision of eHealth. Mobile apps for health have the potential to target heterogeneous populations, but with the ability to also address specific needs and complement highly developed health care technologies. The market is evolving rapidly, generating myriad opportunities for the development of new mobile technologies [6].

Mobile phones (ie, mobile phones with advanced computing and Internet access) and tablet computers (ie, general purpose computers contained in a single panel and usually operated through a touch screen) have become the most popular and widespread types of mobile device [7]. Close to 55% of British adults claim to own a mobile phone [8] and over a third own a tablet [9]. In the United States, a report by the Pew Research Center found that 64% of all adults now own a mobile phone [10] and 34% of American adults own a tablet computer [11]. Worldwide, just under 17% of the 6 billion mobile subscriptions are mobile phone subscriptions [12]. As retail prices decline, ownership of these devices is likely to continue to increase [8] in high-income and low- and middle-income countries.

Mobile apps are increasingly used in managing various tasks in daily life. More than 900,000 apps are available in the Apple App Store (iOS operating system; Apple Inc) and more than 700,000 apps in the Google Play Store (Android operating system; Google). Over 100,000 of these are health-related apps.

Sophisticated computing features mean that both mobile phones and tablet computers can support self-management functions and deliver them at a population level. Self-management interventions could be offered within software extensions that users add to their devices, popularized under the term apps [13].

## Methods

### Overview

We undertook a systematic review of apps used to facilitate self-management of long-term conditions with an outcome focus on the key disease markers and symptoms. The long-term conditions were diabetes mellitus, cardiovascular diseases, and chronic lung diseases. Cardiovascular diseases include hypertension, coronary artery disease, and congestive heart failure. Chronic lung diseases include asthma and chronic obstructive pulmonary disease. We chose these conditions on account of their high global burden [14].

Our definition of self-management apps was software programs designed for mobile phones and tablets that aim to promote or support self-management skills to manage the key disease markers and symptoms. Apps are optional add-ons to the device that interact with users through a set of interfaces (eg, a visual user interface). Health apps can be characterized as a medium with broad capabilities to communicate information, provide interactive experiences, and collect information from patients. They provide a platform for the delivery of self- management interventions that are highly adaptable, of low cost to the health system, and easily accessible.

Using Boolean phrases, we searched PubMed, Embase and EBSCO databases for studies that assessed the effectiveness of apps in the management of diabetes, cardiovascular diseases, and chronic lung diseases. We searched PubMed using Medical Subject Headings and advanced search builder features. Emtree terms using the explosion function to extend the search were used to build a multiterm query along with advanced searches in Embase. We included CINAHL, PsycINFO, and PsycARTICLES in the EBSCO database search. We handsearched the JMIR journals and *Telemedicine Journal and e-Health* as the key publications for this area of research. We examined the reference lists of all papers included in the review and removed duplicates. The databases were searched between 2005 and 2016, since technologies prior to 2005 are unlikely to be representative of contemporary technologies that support health apps [15-17]. In addition, the concept of self-management was not widely adopted prior to 2005. We completed the final search in February 2016 (Textbox 1). The Preferred Reporting Items for Systematic Reviews and Meta-Analyses (PRISMA) statement guided the reporting of the review [18].

**PubMed search strategy (terms).**

1. Pulmonary disease, chronic obstructive
2. asthma
3. Acute coronary syndrome
4. Blood glucose
5. Blood pressure
6. Coronary Disease
7. Cardiovascular diseases
8. Diabetes mellitus
9. Forced expiratory volume (FEV)
10. Hemoglobin A, glycosylated
11. Hypertension
12. Computers, Handheld
13. Peak expiratory flow rate
14. Cell Phones
15. MP3-Player
16. Telemedicine
17. Or/1-16
18. Limit 17 to yr=2005-Current

### Inclusion and Exclusion Criteria

We included original research published in peer reviewed journals that evaluated self-management apps for their effect on disease-specific clinical outcomes. The focus on disease-specific clinical measures such as glycated hemoglobin (HbA_1c_) or blood pressure was chosen because improved clinical outcomes are the ultimate goal (in terms of quality-adjusted life years, disease burden, and health care costs) of self-management programs. Included studies were randomized controlled trials (RCTs) of self-management interventions for patients with a clinician-diagnosed long-term condition delivered via mobile phone apps compared with either self-management interventions delivered via traditional methods (eg, paper-based diaries) or usual care.

We excluded papers if (1) they reported on primary prevention among healthy or at-risk groups, (2) the focus lay outside of the self-management domain (see Textbox 2 for self-management-related activities [3,19]), (3) the sample did not include people living with diabetes, cardiovascular diseases, or chronic lung diseases or where the results from the subsample of the populations of interest were not distinctly reported, or (4) the intervention targeted health care professionals; required modification of hardware; relied solely on messaging (short message service or multimedia message service); did not offer a mode of interaction (this could be automated and based on logarithms), acting only as a transmitter of data (eg, from patient to clinician), because this would be more reflective of telemonitoring; or used devices that did not offer portability comparable with mobile phones and tablets (eg, desktops, laptops, notebooks, and netbooks)—although these are portable, they are not accessible at all times regardless of location. In addition, we excluded review papers, editorials, commentaries, dissertations, poster presentations, abstracts only, proposals for future studies, study protocols, and descriptive papers describing apps but not testing them in a sample population. Publication language was restricted to English only.

**Patients’ self-management characteristics (adapted from Lahdensuo [ 19] and Battersby et al [ 3]).**

- Accept the condition as a long-term disease amenable to intervention
- Have knowledge about the disease and its treatment
- Actively participate in the control and management of the disease
- Identify factors that make the condition worse
- Be able to describe strategies for avoidance or reduction of exacerbating factors
- Recognize the signs and symptoms of deterioration in health
- Follow a prescribed, written treatment plan
- Use correct technique for taking drugs
- Take appropriate action to prevent and treat symptoms in different situations
- Use medical resources appropriately for routine and acute care
- Monitor symptoms and objective measures of disease control
- Identify barriers to adherence to the treatment plan
- Address specific problems that have an impact on the individual’s condition

### Data Extraction and Analysis

We initially screened publications for potential inclusion based on simultaneous review of title and abstract by 2 reviewers. Any discrepancies were resolved by consensus between the reviewers with reference to the full paper. Information extracted from each paper using a structured form included objectives, types of intervention, setting, sample characteristics, outcomes measured, and results reported. We assessed risk of bias for all included studies using the Cochrane Collaboration’s tool for assessing the risk of bias in RCTs [20]. The 2 reviewer authors independently assigned each domain of the Cochrane Collaboration’s tool of each individual study to 1 of 3 categories: low, high, or unclear risk of bias. For each study, we created a risk-of-bias table.

We performed descriptive analyses of the data and summarized the findings from these studies, with emphasis on statistical results reported in RCTs. Differences between groups were highlighted when these results were available. Outcomes were organized into disease-specific clinical outcomes of the intervention. Where available, the usability, feasibility, and acceptability of the intervention were described.

## Results

### Summary

**Figure 1:**
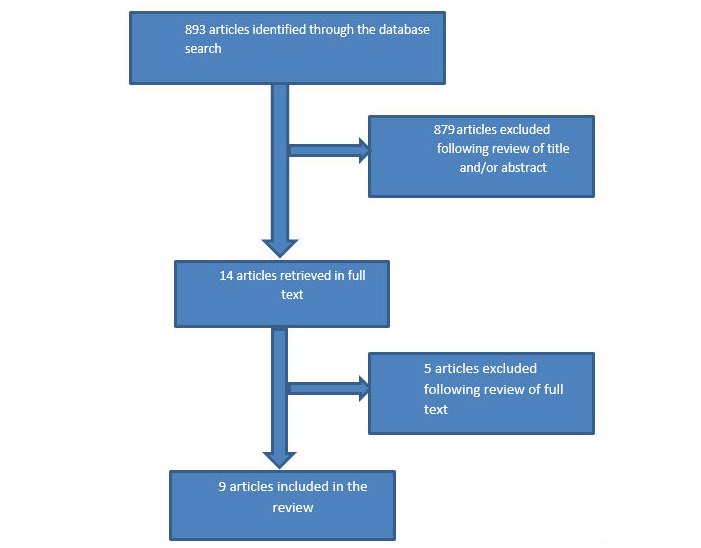
Selection process.

In all, we reviewed the title or abstract, or both, of 893 papers as retrieved by the database searches. We retrieved 14 papers in full text and assessed them for eligibility. We excluded 5 papers because they did not meet the study design criteria. A total of 9 papers met all inclusion criteria. Figure 1 illustrates the selection process. Multimedia Appendix 1 sets out the quality of the included studies, based on the risk-of-bias assessment. The area in which all studies were assigned a rating of high risk was “blinding of participants and personnel (performance bias).” Due to the nature of the intervention, participants could not be blinded in any of the studies. In some of the studies the personnel involved were also aware of to which group participants were assigned.

### Study Characteristics

Publication years ranged from 2008 to 2014 (Multimedia Appendix 2). Studies were included from 4 geographic regions (Europe, n=3; Oceania, n=2; Asia, n=3; United States, n=1). All included studies were RCTs that assessed the effectiveness of interventions that involved mobile phone- or tablet-assisted self-management programs and either standard care (n=8) or offline self-management programs (n=1) [21]. Of the self-management group, 3 included multiple intervention groups using permutations of an intervention involving an app. Charpentier et al [22] ran 2 intervention groups: 1 group used an app plus quarterly clinical visits, and the second used an app plus visits every 2 weeks. Quinn et al [23] ran 3 intervention groups, all involving the patient using an app but with varying degrees of information generated by the app sent to clinicians. Holmen et al [24] ran 2 interventions, 1 using the app alone plus usual care, and 1 using the app and monthly telephone counselling. All 3 involved the use of an app and a Web portal with differing levels of clinician support.

Interventions to address diabetes mellitus (5/9) were the most common, followed by chronic lung disease (3/9) and cardiovascular disease (1/9) interventions. The duration of the intervention ranged from 6 weeks to 1 year, and final follow-up data ranged from 3 months to 1 year. Sample size ranged from 48 to 288 participants.

### Impact on Clinical Outcomes

All 9 studies reported the effect of the intervention apps on disease-specific clinical outcomes (Table 1). Significant differences between groups on the primary outcomes were reported in 6 studies [21-23,25-27]. No significant differences on the primary outcomes were found in 2 studies [24,28,29]. Of the 5 interventions related to improving diabetes management [22-25,27], 2 related to type 1 diabetes [22,25] and 3 related to type 2 diabetes [23,24,27]. Significant improvements in diabetes-specific clinical outcomes (HbA_1c_) were reported in 4 studies [22,23,25,27], 2 relating to type 1 diabetes [22,25] and 2 relating to type 2 diabetes [23,27]. Only 1 study evaluated apps for cardiovascular diseases [29]. Significant improvement in the key clinical outcome, the 6-minute walking test, was observed within the intervention group, indicating overall improved physical functioning, but not between the 2 intervention groups. Mixed results were observed in chronic lung disease clinical outcomes. Lung function parameters were the primary outcomes of interest. Significant improvements were reported in a study on asthma [26] and a study on chronic obstructive pulmonary disease [21], but 1 study, on asthma [28], did not report a significant change.

**Table 1:** Effectiveness of interventions using mobile phone and tablet apps on primary clinical outcomes (n=9).

Long-term condition addressed	Significant effect, n	No significant effect, n	Total n
Diabetes mellitus type 1	2	0	2
Diabetes mellitus type 2	2	1	3
Cardiovascular disease	0	1	1
Chronic lung diseases	2	1	3
Total clinical outcome studies	6	3	9

### Participants

The average age of participants in the studies ranged from 33.8 years [22] to 72.1 years [21]. The age of the participants in relation to ability to use the technology and engagement was not a focus in any study, and none of the studies excluded older people specifically. Holmen et al [24] reported that users >63 years were significantly more likely than younger patients to be substantial users of the app (*P*=.045).

The majority of studies focused on those with more severe symptoms and clinical indicators outside of the normal range. Of the studies on diabetes, 4 focused on those with HbA_1c_levels above the optimal range [22-25], and 2 studies [21,26] focused on people with moderate to severe chronic obstructive pulmonary disease and asthma. The characteristics of the target user group was often the impetus for the development of the app tool or mHealth approach, citing the potential for reduced travel to a medical center and ease of engagement as incentives for self-management for these groups with greater symptom burden or higher risk of burden in the future.

In 1 of the studies [25], participants had to provide the phone (and presumably also the network connection) and in 1 study [28], the participants were required to have a contract with a compatible network at their own cost, though a phone could be provided. Neither study noted any significant barriers in recruitment as a result of this inclusion criterion. In all other studies the equipment and the network setup and costs were covered by the research study.

### Interventions

The interventions differed in two main ways: the combination of tools used in the intervention and the level of clinician input (Multimedia Appendix 1). Thus, 2 studies [26,28] used an app only and 3 studies ran 2 or 3 intervention groups, 1 of which involved the use of an app only [22-24]. A total of 3 studies [22,23,26] demonstrated a significant change in symptom management and 2 [24,28] did not. An app plus feedback or contact with participants, either by text (4 studies) or phone conversation (3 studies), was used in 7 interventions. A total of 7 studies also used automatic text messages generated by the app data, but only 2 studies [26,28] used this form of feedback alone. In interventions that involved additional clinician input, only 1 study standardized the enhanced clinical input across the control group and intervention [26]. In 6 studies the level of clinician input and support for the intervention group was enhanced. In these studies, the effect of using an app cannot be isolated, and the results cannot be interpreted in relation to increased clinician input or support and the use of an app.

Of the 5 studies that explored the impact of using an app only as the intervention tool on clinical outcomes [22-24,26,28], 3 studies [22,23,26] demonstrated a significant change in symptom management and 2 [24,28] did not. Only 1 study isolated the contribution of the app intervention where clinical care was standardized for the intervention and control group [28]. The study did not report a significant change in asthma symptom control, with symptom control improving marginally in both groups. Similar to the latter study, 4 studies standardized clinical care, with both the control and intervention groups invited to attend one outpatient appointment every 3 months [22-24,26], as per best practice. However, a difference between these 4 studies and the study by Ryan et al [28] was the sharing of app data ahead of the clinic visit [23,26] or the option of sharing the app data during the scheduled clinic visits, in anticipation that the data collected over time would inform the consultation and medical management [22,24]. The impact of this is difficult to quantify. In 2 studies [22,24], sharing data was by the patient’s choice, and the number of patients who chose to do so was not reported. In a study involving 2 intervention groups, the effectiveness of the app-only intervention was significant, although the effect size for the app-only intervention group was not as big as that of the intervention group involving an app and teleconsultations [22].

Another study [24] demonstrated an improvement in HbA_1c_in all 3 groups (a control and 2 intervention groups), although the improvement was not significant for any group. Interestingly, HbA_1c_decreased more in the app-only group (0.31) than in the control group (0.16) and in the intervention group (0.15) that also involved counselling. In a study on asthma [26], the data were sent to the clinician ahead of the patient consultation, but no data were reported on whether the clinician used this. Only 1 study [23] specifically sought to explore whether sending clinicians’ data improved clinical outcomes. All 4 groups continued with usual care (a review with their primary health care provider every 3 months); however, intervention group 1 participants could choose to share their app-generated data with their provider. For intervention group 2 participants, the clinician was sent unanalyzed app-generated data before the scheduled appointment. For intervention group 3 participants, the clinicians were sent analyzed app-generated data ahead of the scheduled appointment. What is not known is how many patients in intervention group 1 shared their data with their clinicians, nor how many clinicians viewed and used the data for participants in groups 2 and 3 in their consultations. The outcome data are mixed, with a decrease in HbA_1c_noted for all 4 groups, and significant differences noted between the control group and intervention groups 1 (*P*=.027) and 3 (*P*=.001), but not for intervention group 2 (*P*=.40).

### Safety Mechanisms

Only 3 studies [22,27,28] reported an inbuilt safety mechanism for the app intervention, whereby a reading outside of the normal range and considered aberrant enough according to the inbuilt logarithm would trigger an alert. In 1 study [28], the alert would be followed up by the asthma nurse linked with the study. In another [27], an email was generated and sent to the principal investigator and the research nurse. A further study noted the parameters that would be considered abnormal but did not state when or how readings outside of the normal range would be followed up or to whom they would be sent [22]. No study described whether cover included an out-of-hours service; 1 study reported that the data did not trigger any alerts [27]; and the other 2 did not comment on the need for follow-up due to abnormal readings [22,28].

Importantly, no study reported an increase in the number of adverse events or need for additional hospital visits or medical care as a result of participating in the interventions.

### Training in the Use of Technology

A total of 5 studies [24,26-29] described training participants in the use of the equipment and input of data. This ranged from distance support [28] with a follow-up 1 week later, to face-to-face support [24,29], and face-to-face support and a 2-week trial as to whether potential participants could use the technology before they were included in the study [27]. Training was mentioned by another study [26] but the nature of this was unclear. In 4 studies participants were offered ongoing technological support, if required, by telephone [24,27-29].

### Technological Issues

Few technological issues were reported. Of the studies, 7 required participants to enter the data generated by the study equipment into the app (or via the website) and 2 used a wireless or Bluetooth-compatible device to transmit the data automatically without requiring the participant to manually submit the data [22,24]. No study reported on erroneous imputations (by participants) and only 1 study [24] reported errors in the transfer of data due to issues with the Bluetooth pairing required for automatic transmission of data from the glucometer to the app in the mobile phone.

No study reported on the number of calls participants or clinicians made for technological support during the study. However, 1 study did note that some participants who travelled overseas incurred high mobile costs that weren’t covered by the research study. The authors noted that this was anticipated, and participants were informed about the different network rates if travelling.

### Usability, Feasibility, and Acceptability

Only 1 study sought to explore the usability and feasibility of the app from the participants’ perspective [27], and 1 study explored acceptability of the app from the health care provider’s perspective [22]. All studies reported on attrition, which provides some indication of the usability, feasibility, and acceptability of the intervention. The attrition rates ranged from 8.75% [22] to 26% [25]. Most studies reported the attrition rate for the control and intervention groups combined. Where the attrition rates were reported for the control and intervention groups separately, of note, in 1 study, the attrition for the intervention group was higher, with the study average at 25.83%, but the intervention group alone was at 28.33% [26]. In another study, the dropout rate in the usual-care group was considerably higher (36.49% vs 13.21%) [29]. The usual-care group were enrolled in a cardiac rehabilitation program. In a third study [23] the attrition rate was as high as 31.82% in 1 of the intervention groups (control group, 21.45%). The average attrition rate across all 4 groups was 25.6%. No study noted any differences by demographics or clinical parameters between those who dropped out and completers.

A total of 3 studies reported on the number of participants who dropped out specifically because of the technology or because the frequency of input was too burdensome (6/17 [26], 3/7 [29], and 2/3 people [27]).

Only 1 study reported that potential participants were excluded because they could not use the technology [27]. In this study, 12 people were specially excluded before random allocation because they were experiencing difficulties using the devices and sending data. This equated to 18% of the group initially recruited into the trial.

A total of 5 studies explored engagement with the intervention over time and described these findings as a proxy for the usability, feasibility, and acceptability of the intervention [22,24-26,29]. In general, the studies found that the app or intervention was usable, feasible, and acceptable to users. The frequency of data entry was noted to decrease over time in 2 studies [24,25], and there was no significant relationship between level of engagement and change in HbA_1c_. Another 2 studies reported adherence at the end point of the study as evidence of acceptability (76.7% of the control group and 71.7% of the intervention group [26]; 46.67% of the control group and 80% of the intervention group [29]). A further study asked participants whether they wanted to continue with the intervention if this were possible at the end of the study, reporting that 67% of participants in the app-only intervention and 75% in the app and teleconsultation intervention confirmed that they would [22]. This study was the only one to report on the health care provider’s perspective, reporting that 77% of health care providers were satisfied or very satisfied (total number of health care providers unknown). This study found that the time spent by clinicians engaging with participants across the groups was the same (average 71 minutes) but the time saved by the intervention group that did not include hospital clinic visits was 281 minutes (average travel time to attend 2 clinic visits). The time taken to upload data (wirelessly) was estimated to be 10 seconds per day.

How participants felt about the time the intervention took and how easily they had incorporated data entry into their daily life was explored in 1 study [27]. Of 24 participants in the intervention group, 14 felt that the daily data entry was easily incorporated and 10 did not. The average time spent entering data was 22.5 minutes per day. Compliance rates over time (from baseline to the last 2 weeks of the study) for the components of the intervention differed, with a 70% compliance rate (daily entry) for morning measurements, 50% compliance for bedtime measurements, and 51.2% compliance related to uploading a photo of a meal.

No study reported on the features of the app that patients or health care providers found useful (eg, automated reminders, text messages with educational and motivational content, increased awareness), but 1 study did report that patients expressed feeling reassured, knowing that their health symptoms were regularly monitored [27]. Although the patients’ perspective wasn’t reported, 2 studies did find that the time saved by not having to visit the hospital for follow-up as per usual care was considerable [22,29].

### Costs

The cost implications of using technology were considered in 2 studies. In 1 study where the participant provided the phone and the network connection and a free app was used [25], the cost to the study of messaging participants was calculated at A$290.93, which equated to A $8.08 per participant (n=36). The intervention also included input from a diabetes educator. The diabetes educator spent on average 3 hours per week reviewing participants’ logs and text messaging participants, equating to 5 minutes per participant, per week (72 hours in total over the 6-month period). With the hourly rate of A$28.85, the cost to the study was A$2077.20.

The second study required the participant to provide the phone and cover network costs. The technological support service for participants and nursing cover (the safety mechanism for abnormal readings) were contracted out to the company that developed the software, and this was the only cost incurred (not disclosed).

## Discussion

### Principal Findings

The evidence presented indicates the potential of apps in improving symptom management through self-management interventions. Of the 9 studies, 6 reported a statistically significant difference in the primary clinical outcome of interest. Where the intervention comprised an app only, 3 studies demonstrated a statistically significant improvement. The interventions differed in two main ways: the combination of tools used in the intervention and the level of clinician input (Multimedia Appendix 1). A total of 2 studies [26,28] used an app only and 3 studies ran 2 or 3 intervention groups, 1 of which involved the use of an app only [22-24]. Symptom management changed significantly in 3 studies [22,23,26] but not in 2 [24,28]. An app plus feedback or contact with participants, either by text (4 studies) or phone conversation (3 studies), was used in 7 studies; 7 studies also used automatic text messages generated by the app data, but only 2 studies [26,28] used this form of feedback alone. In interventions that involved additional clinician input, only 1 study standardized the enhanced clinical input across the control group and intervention [26]. In 6 studies the level of clinician input and support for the intervention group was enhanced. In these studies, the effect of using an app cannot be isolated, and the results cannot be interpreted in relation to increased input or support and the use of an app.

Given the evidence that monitoring alone improves symptom control [30], in our review we sought to understand the contribution an intervention involving an app can make. Separating the effect of monitoring alone was not possible in this review. None of the interventions included a study group that involved telemonitoring only. All of the interventions involved either real-time automated feedback or clinician-initiated feedback based on the data entered. The second issue confounding the findings regarding the contribution of the app to symptom management was the use of additional interventions, in combination with the app, to support symptom management.

A further note of caution relates to the ability to generalize the findings to the clinical setting. All of the studies referred to clinical care during the study period as relating to best practice and may indicate an improvement in the actual usual care received. Aside from 1 study [28] where a nurse was employed to ensure follow-up appointments every 3 months, the clinical follow-up appears to have been undertaken in real-life, usual-care clinical settings, although the prompt for data collection at the 3-month time points may have increased the likelihood of these appointments being scheduled and kept. In 1 study [28] the authors specifically noted that, although the clinical care provided during the study period was set up to be in line with best practice with 3-month follow-up appointments, this was likely an improvement in clinical support for the study participants and that it remains unknown whether the addition of the app could improve clinical outcomes when clinical care is less than optimal.

A total of 7 interventions involved the use of the app and a degree of clinical input or support. It does not appear that the intensity of support necessarily affected the outcome, nor the mode of support (eg, electronic, verbal, or face to face). All of the interventions that involved weekly support did note a significant improvement in symptom control, and interventions with a greater gap in time between contact noted mixed results. However, interventions without additional clinical input between usual clinical visits also showed a significant improvement. More frequent clinical input or engagement does not appear to be vital in effecting change in symptom management.

All studies reported minimal issues related to usability. It appears that apps can be used by those with little technology experience or familiarity. The apps do, however, rely on the active engagement of the user. In this review, we could not discern how frequent the level of engagement between user and app needs to be and for how long in order to effect long-term change in symptom management. The engagement of health care providers in monitoring symptoms and exchanging information with users would be desirable in terms of promoting partnership in care, although, again, how important this is in effecting improvements in symptom management is not clear from the review. The freedom and portability of mobile devices, combined with the advanced capacity to facilitate 2-way communication and collect and analyze data for a real-time response, offer enormous potential to patients and health care providers. The potential complexity of today’s mHealth tools and the mixed evidence on the features that are important and make a difference in their effectiveness indicate a need for a focus on understanding the connection between patient experience, adherence, and health outcomes.

The involvement of end users in the development of apps and also specific groups, such as older people and those from different cultures, was not a strong feature in the interventions reviewed. An iterative design process involving systems and content development and multiple stages of user experience testing is recommended for future apps aimed at similar patient populations [31]. The wider evidence suggests that diverse groups can use apps with sufficient training and provision of support [4,32,33], although the level of support over time in both areas remains relatively unexplored.

Technical problems did not feature highly in the review. The issue of erroneous imputations was not mentioned, and errors in transfer of data was noted in only 1 study. However, these areas have been raised in other studies as requiring regular monitoring and attention [34,35].

Given the multitude of apps available, advice on how to develop a “good” app or assess the “quality” of an app when reviewing the existing apps available has important ethical and legal issues for both research and clinical practice. The market has low entry barriers, and ease of accessibility for users through mobile phones and tablets makes this an attractive area for both private and professional areas of application. In health care, when mobile smart devices are used in combination with add-ons that are connected either directly or via wireless technology—for example, blood glucose monitors—manufacturers are required to conform to the laws and regulations that are in place for medical devices, although, depending on the jurisdiction, apps may or may not be well adapted to the specifics of mobile devices. Regulation usually encompasses an app running on a smart device and, for the health professional and patient, some reassurance as to the level of trustworthiness. Stand-alone smart devices and the apps running on them may pose a significant threat to a patient’s safety and privacy if the necessary safety measures are not observed. Before recommending or developing an app, it is vital that the functionality be thoroughly tested with respect to the potential for miscalculations, erroneous or incomplete content, technical deficiencies, and other usage restrictions. In addition, assessing the validity of the information and advice regarding symptom management is vital, as well as safety mechanisms relating to when to seek urgent medical advice and support.

The issue of adherence over time was reported by some studies in the review and was found to be unrelated to clinical outcome, that is, those who continued to input data and those who were classified as substantial users did not differ from those whose input declined over time. However, the issue of maintaining engagement over the course of a study and beyond into everyday life is a major consideration and highly likely to affect long-term symptom control. There is a growing understanding of barriers to adherence and ways to overcome them. The development of mAdherence tools to explore barriers to maintaining engagement is growing and will be important in the development of mHealth interventions. In this review we noted differences in patient-provider communication and in the use of targeted motivational messages, but we were not able to qualify their impact.

The issue of enhancing adherence goes beyond maintaining engagement of patients using apps. The development of mHealth tools for chronic disease management could unintentionally increase health disparities in access to technology. Vulnerable, hard-to-reach, or otherwise high-risk patient populations run the risk of exclusion. Where mHealth tools have the potential to engage patients who are less inclined to use traditional health services, mHealth tools offers a way to address barriers to care and reduce health disparities. It is important that future studies specifically build on these areas and reduce the risk of generating a range of interventions largely unused by those who could benefit the most.

Few of the studies we reviewed discussed the issue of cost. The majority of the studies provided the devices to study participants. When implemented at scale, interventions that use patients’ existing mobile devices rather than relying on gifted devices will go further toward explaining feasibility and improving clinical outcomes. Rigorous cost-effectiveness analyses will be necessary to demonstrate not only the health impact, but also the value of investing in these innovations.

In addition to cost barriers, other potential barriers for consideration are language and literacy barriers, as well as availability and connectivity issues. Perhaps most critically, if adherence to chronic disease management is not encouraged and actively practiced, it is unlikely that mHealth tools, which are communication platforms and delivery mechanisms, not solutions in and of themselves, will be effective. Conditions such as capability, opportunity, and motivation are essential to behavior change [36]. For example, in diabetes, to improve HbA_1c_, management of healthy eating, physical activity, and adherence to medication are all important, yet we know little about how we can support and effectively motivate a person in all 3 areas through an app. This again points to the need in future research for the involvement of users as part of the team when developing interventions.

### Limitations

There are limitations to this systematic review. A meta-analysis was not possible due to the heterogeneity of the study designs. We did not include non-English literature. The diversity of study objectives, designs, and outcomes made clear comparisons difficult, and the quality of evidence was variable.

Our review expands the evidence base by extending the definition of app interventions to include interventions integrating apps, by assessing both clinical and self-management outcomes, and by contributing to the emerging literature regarding mHealth feasibility, usability, and acceptability.

### Conclusion

The use of apps in mHealth has the potential to improve health outcomes among those living with chronic diseases through enhanced symptom control. Further evaluation of apps used in mHealth, and more widely in eHealth, will be valuable. Research that involves populations traditionally marginalized and research into how these tools can help to overcome barriers to chronic disease management will be especially relevant. Further innovation, optimization, and rigorous research around the potential of apps in mHealth technology will move the field toward the reality of improved health care delivery and outcomes.

## Appendix

Multimedia Appendix 1 Risk of bias assessment for included studies.

Multimedia Appendix 2 Sample characteristics and intervention.

## Abbreviations

HbA1c: glycated hemoglobin
PRISMA: Preferred Reporting Items for Systematic Reviews and Meta-Analyses
RCT: randomized controlled trial
