# Supplementary material for: The Effectiveness of Self-Management Mobile Phone and Tablet Apps in Long-term Condition Management: A Systematic Review
Source: J Med Internet Res. 2016 May 16;18(5):e97. doi: 10.2196/jmir.4883 (PMC4886099; doi:10.2196/jmir.4883)
Supplement: Multimedia Appendix 1 [file jmir_v18i5e97_app1.pdf]

Appendix I. Risk of bias assessment for included studies.

Liu et al 2008 [21]

Ryan et al 2012 [28]

Charpentier et al 2011 [22]

Risk of bias

Authors' judgement

Support for judgement

Authors' judgement

Support for judgement

Authors' judgement

Support for judgement

Bias

Low risk

Random number table used to assign participants.

Low risk

Central telephone randomisation provided

by the University of Aberdeen's Health Services

Research Unit

Unclear risk

Authors state that participants were randomised, using a web based system but do not state who completed and how this was achieved.

Random sequence generation (selection

bias)

Unclear risk

Not enough information available in the study report

Low risk

Telephone-controlled randomisation

Unclear risk

Not enough information available in the study report

Allocation concealment (selection bias)

High risk

Due to the nature of the intervention, participants and personnel could not be blinded.

High risk

Blinding of participants or personnel was not possible due to the nature of the intervention. This could have affected participants' performance

High risk

Due to the nature of the intervention, participants and personnel could not be blinded. This may have affected performance.

Blinding of participants and personnel

(performance bias)

Unclear risk

Not enough information available in the study report.

Unclear who collected the data at the clinical visits.

All outcomes

Low risk

Researchers blinded to allocation. Collected data on primary outcomes at the final trial visit

Low risk

Data were recorded electronically. Two of the groups received face to face visits at months 3 & 6 (unclear, but appears to be with medical staff and not the research team) and one group engaged in fortnightly teleconferences.

Blinding of outcome assessment (detection

bias)

Low risk

Six participants in each group withdrew from the study. The sample size at follow-up still met the sample size required to detect a significant difference according to the power calculation.

All outcomes

Unclear risk

Data appear to have been analysed using an available case analysis

Low risk

Some attrition noted (N=7) but a substitution method was used for the primary outcome measure.

Incomplete outcome data (attrition bias)

Low risk

All variables reported in the methods were reported upon.

Low risk

Authors reported the outcomes and performed

the statistical analyses they had listed in the study protocol.

Low risk

All intended outcome variables were reported upon.

Selective reporting (reporting bias)

Unclear risk

It also cannot be guaranteed that the patient was actually walking during the whole duration of music played.

High risk

The study duration was 6 months however recruitment across all the GP practices was rolled over 13 months. This could have introduced a confounder in the form of a seasonal effect of asthma. Authors did not have the means to make allowances for a potential seasonal effect.

High risk

The amount of contact varied between the groups and this could have affected the outcome. This was not controlled for.

Quinn et al 2011 [23]

Holmen et al 2014 [24]

Kirwan et al 2013 [25]

Bias

Authors’ judgement

Support for judgement

Authors’ judgement

Support for judgement

Authors’ judgement

Support for judgement

Random sequence generation (selection

bias)

Unclear risk

Primary care practices were randomised to one of four

study groups using a stepped intervention design for groups. Unclear what programme was used and who completed the process.

Low risk

Randomisation was achieved through block-randomization and conducted at the Center of Randomization at the Unit for Applied Clinical Research at the Norwegian University of Science and Technology in Trondheim, using WebCRF (Case Report Form).

Low risk

The study coordinator randomized patients using a freely available online randomization program. A permuted block randomization design method was used during the 3-month rolling recruitment to ensure roughly equal numbers of patients were allocated to each comparison group

Allocation concealment (selection bias)

All outcomes

Unclear risk

Not enough information available in the study report.

Unclear risk

Not enough information available in the study report.

Low risk

Automated, computerised process.

Blinding of participants and personnel

(performance bias)

High risk

Due to the nature of the intervention, participants and personnel could not be blinded.

High risk

Due to the nature of the intervention, participants and personnel could not be blinded.

High risk

Due to the nature of the intervention, participants and personnel could not be blinded. This may have affected performance.

Blinding of outcome assessment (detection

bias)

Unclear risk

Not enough information available in the study report.

Researchers collected the secondary follow-up data at baseline, 3, 6, & 9 months. Unclear whether they were blinded.

High risk

The research team was involved in the assessment of eligibility, data collection, training of patients to use the devices, and follow-up.

Thus, those who delivered technical support had to know which group the participants were allocated to.

Low risk

There was no face to face contact between the researchers and participants in relation to data collection-completed electronically and via the labs (for HbA1c). Data collected was comparable between the intervention and control groups.

Incomplete outcome data (attrition bias)

Low risk

Intention to treat analysis completed.

Low risk

The sample size at follow-up still met the sample size required to detect a significant difference according to the power calculation. After the 1-year follow-up, there was a total dropout attrition rate of 21% (31/151), with an equal distribution in the groups.

Baseline analysis revealed no difference between those lost to follow-up and those who completed the study for all variables.

High risk

Dropout was 26% (11 males, 8 females, 19/72) with logistic regression analysis revealing no significant difference in age, gender, diabetes duration, insulin pump use, and baseline HbA_{1c} among those that completed the study and those that were lost to follow up. However, this may have caused a type 2 error (potentially underpowered to detect differences on secondary outcomes).

Selective reporting (reporting bias)

Low risk

All intended outcome variables were reported upon.

Low risk

All intended outcome variables were reported upon.

Low risk

All intended outcome variables were reported upon.

Other bias

Low risk

None noted.

Low risk

None noted.

Unclear risk

This study was a randomized controlled trial with a small sample conducted over a short duration. Due to the dropout of patients, the study may not have been powered sufficiently to detect differences between groups for the secondary outcome measures. There were differences in glycaemic control and gender between groups at baseline. Although patients in the control group were instructed not to use any mobile applications to self-manage their diabetes during the study period, it is possible that they did.

Liu et al 2011 [26]

Varnfield et al 2014 [29]

Waki et al 2014 [27]

Bias

Authors' judgement

Support for judgement

Authors' judgement

Support for judgement

Authors' judgement

Support for judgement

Random sequence generation (selection

bias)

Unclear risk

Not enough information available in the study report

Low risk

Permuted-block randomisation, by computer-generated random numbers with variable block sizes of 4, 6 and 8 using sequentially numbered opaque, sealed envelopes, was conducted prior to baseline assessment to randomise patients to one of two parallel groups: control (TCR) and intervention (CAP-CR). The project officer enrolled and

assigned participants, administered baseline questionnaires and

notified the CR clinician to book a baseline assessment.

Low risk

Computer generated list of random numbers employed to randomise.

Allocation concealment (selection bias)

All outcomes

Unclear risk

Not enough information available in the study report

Low risk

Sealed envelopes used to assign participants according to randomisation.

Unclear risk

Not enough information available in the study report.

Blinding of participants and personnel

(performance bias)

All outcomes

High risk

Neither participants nor personnel could have been blinded due to the nature

of the intervention. This is likely to have affected participants' performance.

High risk

Due to the nature of the intervention, participants and personnel could not be blinded.

High risk

Due to the nature of the intervention, participants and personnel could not be blinded.

Blinding of outcome assessment (detection

bias)

Unclear risk

Not enough information available in the study report

Unclear risk

It is unclear how the secondary follow-up data was collected (by mail, by completion at clinic appointment) and whether a researcher was present.

High risk

The researchers collected the data and were not blinded as to allocation.

Incomplete outcome data (attrition bias)

Unclear risk

Not enough information available in the study report

Low risk

Intention to treat analysis completed.

Low risk

Intention treat analysis

Selective reporting (reporting bias)

Unclear risk

Not enough information available in the study report

Low risk

All intended outcome variables were reported upon.

Low risk

All intended outcome variables were reported upon.

Other bias

Unclear risk

The study duration was 6 months. Since participants were recruited from different

outpatient clinics it is likely that recruitment of participants spanned over a longer period. Therefore, there could have been a seasonal effect of asthma acting as a confounding

variable

Low risk

A limitation is the small sample size. Although

powered for completion rate outcome, it was too small to demonstrate functional capacity outcomes, particularly due to considerable dropouts. CAP-CR reduced waiting time from referral to commencing CR by 2 weeks on average. The shorter recovery time may have influenced outcomes. This study did not

observe the effect of this outcome between the two groups.

High risk

The size and duration limit the generalizability of the results. Participants were to input all their meals (including photos) for dietician evaluation. The majority of patients input only 1 meal per day, 2 at best, reporting that the procedure took too long.

As a result, this impacted on understanding of how dietary habits improved over the study period, and

how those habits affected diabetes control. The usability survey was developed specifically for this study and was not a validated questionnaire.
