# Supplementary material for: The Effectiveness of Self-Management Mobile Phone and Tablet Apps in Long-term Condition Management: A Systematic Review
Source: J Med Internet Res. 2016 May 16;18(5):e97. doi: 10.2196/jmir.4883 (PMC4886099; doi:10.2196/jmir.4883)
Supplement: Multimedia Appendix 2 [file jmir_v18i5e97_app2.pdf]

Appendix II. Sample characteristics and intervention.

Study	Age (mean)	Gender	Time since diagnosis	Special considerations	Intervention	Intervention length
Charpentier et al (2011) [22]	33.8 years (SD 12.9)	66 male, 114 female	Unknown	Participants were over 18 years old, had type 1 diabetes for at least 1 year, and had been treated with a basal bolus insulin regimen for at least 6 months, either with MDI or with a pump. They were eligible for the study if their last HbA1c values during the year before and at entry of the study were ≥8.0%.	**App and hardware:** Participants in the intervention groups received a smartphone loaded with the Diabeo software. Diabeo software is a bolus calculator with validated algorithms (5), taking into account SMPG level before meals, carbohydrate counts, and planned physical activity. Parameters personally tailored for adjustment of prandial and basal insulin dose were entered into the system for each patient. If fasting or postprandial SMPG do not meet target levels, the system can suggest adjustments for carbohydrate ratio, long-acting insulin analog dose, or pump basal rates. Diabeo software was edited by Voluntis (Paris, France), in collaboration with CERITD.<br><br>**Frequency of data input by participant:** Participants were encouraged to enter at least two blood glucose readings daily.<br><br>**Real-time feedback:** Yes, for insulin calculation dose.<br><br>**Additional software:** Data could also be viewed on a web platform<br><br>**Input from Clinician:** Control group and intervention group 1: hospital visit for review at 3 and 6 months. Clinicians could access the patient data via the web portal or smartphone. For	6 month intervention and 6 month follow-up.

					intervention group 2, no hospital visit, instead, **w**eekly text message feedback from a Certified Diabetes Educator for the first 6 months of the study.	
Holmen et al (2014) [24]	57.3 years	89 male, 62 female	Unknown	Patients with type 2 diabetes HbA1c above the national recommendation (HbA1c>7.0%, 53 mmol/mol)	**Control group:** Advised to continue with usual care which involved a minimum of an annual visit to the GP for HbA1c measurement.<br><br>**Intervention groups:** Participants in both intervention groups received a mobile phone with the Few Touch Application (FTA) self-management system. The FTA system provided the user with a diabetes diary app designed to increase self-management through awareness, overview of relevant factors, and motivational feedback through symbols such as smiling faces and colour codes in the app. The participants measured blood glucose levels with a glucometer (LifeScan OneTouch Ultra Easy), which enabled automatic transfer of the measurement to the diary mobile app through a wireless Bluetooth connection and provided visual graphs, trend reports, and feedback through colour coding (below normal, normal, and above normal). The app also consisted of a food habit registration system, a physical activity registration system, a personal goal-setting system, and a general information system. The user entered information about food intake, physical activity, and personal goals. The second intervention group involved the use of the FTA as above and patient received monthly counselling sessions via phone with a diabetes specialist	4 month intervention, 12 month follow-up.

					nurse.<br><br>**Frequency of data input by participant:** Unclear how often patients were asked to enter data. Participants were classified as substantial and non-substantial users.<br><br>**Real-time feedback:** Yes, in the form of graphs and symbols such as smiling faces and colour codes.<br><br>**Additional software:** Unclear whether a web portal/platform was also used.<br><br>**Input from Clinician:** All groups continued with usual care (GP visit at least annually). Patients in the FTA and counselling group received clinician input on a monthly basis only.	
Kirwan et al (2013) [25]	35.2 years	28 male, 44 female	18.94 years	Type 1 diabetes. Patients with HbA1c above the national recommendation (HbA1c>7.5%).	Intervention participants, asked to download the app "Glucose Buddy". This is freely available app. Users can enter blood glucose levels, insulin dosages, other medications, diet (food items in grams), and physical activities (in minutes). Users can view their data on a graph and export the data via email. A website was also available through which that patients could enter and view their data.<br><br>**Frequency of data input by participant:** No minimum or maximum level of input stipulated.<br><br>**Real-time feedback:** Patients could click on graph option to see plot of results but no real-time messages or feedback.<br><br>**Additional software:** Also able to input data and review results via the website.	6 month intervention. 9 month follow-up.

					**Input from Clinician:** The information was reviewed weekly by a clinician using the web interface. Weekly text message feedback from a Certified Diabetes Educator for the first 6 months of the study.	
Liu et al (2008) [21]	72.1 years	24 male, 24 female	Unknown	Moderate to severe COPD, specified ≤40 years and ≤80 years	**Control group: C**linic visit every month for the first 3 months. Fortnightly telephone reinforcement/support re completing the walking exercise at home.<br><br>**Intervention group:** The software used was a Java application (Java 2 Micro Edition (J2ME)) designed by the National Centre for High-Performance Computing (Hsinchu, Taiwan). It is compatible with most<br><br>commercial J2ME-enabled cell phones with General Packer Radio Service (GPRS). The present study adopted the Sony Ericsson K600i*1* cell phone (Sony, Tokyo, Japan) to execute the J2ME application for the endurance walking exercise by music pacing and data uploading through GPRS to a website.<br><br>**Frequency of data input by participant:** Once daily.<br><br>**Real-time feedback:** Feedback was available immediately on the website, unclear whether this could be accessed by the smartphone or whether patients had to log into the website to access this.<br><br>**Additional software:** Web platform<br><br>**Input from Clinician:** Both groups, clinic visit every month for the first 3 months. Both groups received	3 month intervention, 12 month follow-up.

					fortnightly telephone reinforcement/support re completing the walking exercise at home.	
Liu et al (2011) [26]	52 years	44 male, 45 female	Unknown	Moderate to severe asthma as defined by the American Thoracic Society [19] on the basis of clinical<br><br>symptoms and physical examination. Patients were treated<br><br>according to their current severity level by the Global Initiative<br><br>for Asthma (GINA) guidelines	**Control group:** 3 monthly outpatient appointment as per usual care.<br><br>**Intervention group:** Mobile telephone-based interactive self-care software created under the collaboration between the Taiwan Chest Disease Association and National Centre for High-Performance Computing, Hsinchu, Taiwan. The self-care software provided an electronic diary to record patients' daily asthma symptom score (sleep quality, severity of coughing, difficulty in breathing and daily activities affected by asthma), use of relievers, peak expiratory flow rate (PEFR) and PEFR variability.<br><br>**Frequency of data input by participant:** Once daily.<br><br>**Real-time feedback:** Yes, assessment of asthma status and management advice based on this.<br><br>**Additional software:** Web portal for accessing data that patients and clinicians could access.<br><br>**Input from Clinician:** Both groups attended an outpatient appointment every 3 months as per recommended care. For the intervention group, clinicians reviewed the data collected between visits to inform the review.	6 month intervention, 6 month follow-up
Quinn et al (2011) [23]	52.93 years	81 male, 82 female	Unknown	Physician diagnosis of type 2 diabetes for ≥months;	**Control group:** Usual care-3 monthly appointment with primary healthcare provider.<br><br>**Intervention groups:** 3	12 month intervention with 12 month

	Glycated haemoglobin ≥7.5% within 3 months; Age 18–64 years	intervention groups. Patients received a One Touch Ultra 2 (LifeScan, Milpitas, CA) glucose meter and supplies. Patients in the three active treatment groups received identical study materials: mobile phones, 1-year unlimited data and service plan, study mobile diabetes management software, and access to the web-based patient portal. The patient-coaching system included a mobile diabetes management software application and a web portal. The mobile software allowed patients to enter diabetes self-care data (blood glucose values, carbohydrate intake, medications, other diabetes management information) on a mobile phone and receive automated, real-time educational, behavioural, and motivational messaging specific to the entered data. The patient web portal augmented the mobile software application and consisted of a secure messaging centre (for patient provider communication), personal health record with additional diabetes information (e.g., laboratory values, eye examinations, foot screenings), learning library, and logbook to review historical data.<br><br>**Frequency of data input by participant:** Unknown<br><br>**Real-time feedback:** Yes, automated messages classified as educational, behavioural, and motivational.<br><br>**Additional software:** Yes, a web portal specifically for the study.<br><br>**Input from Clinician:**	follow-up.

					Diabetes educators acted as virtual case managers and intermittently reviewed the data and could supplement automated messages with messages sent to the patient portal. Patients in all intervention groups could call the educators but were encouraged to contact them, if required, electronically. Patients in the intervention group were sent an electronic action plan every 2.5 months summarising their activity. The clinicians linked to patients in the intervention groups received different levels of training and access to the information on the portal.	
Ryan et al (2012) [28]	49.05 years	163 male, 125 female.	Unknown	fulfilled the criterion of poorly controlled asthma<br><br>12 years and over.	**Control group:** Clinical care based on best practice (3 monthly clinic visits).<br><br>**Intervention group:** Eligible patients either had a contract with a compatible mobile phone network and a compatible handset or patients with an incompatible handset but who subscribed to a compatible network were offered the opportunity of borrowing a handset for the duration of the trial. The t+ Asthma application (OBS medical) was loaded onto the phone and functionality tested. The t+ Asthma application enabled twice daily recording and transmission of symptoms, drug use, and peak flow.<br><br>**Frequency of data input by participant:** Twice daily<br><br>**Real-time feedback:** Yes, the recorded peak flow was displayed within the traffic light zones and patients encouraged to follow their action plan.	6 months with a 6 month follow-up.

					**Additional software:** Data could be accessed by the patient and clinician via a password protected website.<br><br>**Input from Clinician:** Both groups received a 30 minute education session on asthma management at the start of the study. Clinical care was standardised for both groups with monthly visits to the asthma nurse until control had been achieved. Safety mechanism: An asthma nurse would contact a patient if peak flow entered the amber or red zone.	
Varnfield et al (2014) [29]	55.6 years	82 male, 12 female.	Unknown	Post MI Average days from cardiac event to commencing a programme were 68 days for TCR and 54 days for CAP-CR.	**Control group:** Usual care=traditional cardiac rehabilitation. This involved hospital visits 3 times a week (1 hour education and 2x 1 hour exercise sessions) over a 6 week period.<br><br>**Intervention group:** Participants entered data daily (via the App) and received 2-4 text messages per day. Mentors provided weekly telephone consultations over 6 weeks, average 15 minutes based on patients' data.<br><br>Each participant in the intervention group, was equipped with a smartphone (Nokia N96, Nokia Inc) preinstalled with health diary (WellnessDiary, Nokia Research) and activity monitoring (StepCounter, Nokia Research) applications; blood pressure (BP) monitor (AXIS Pacific C/-Delmond flexibles Pty Ltd); and weight scale (Glass Body Analysis scale, Propert). Activity monitoring (step number, duration and intensity) was automatic through the phone's	6 week intervention with 6 month follow-up.

					in-built accelerometer.<br><br>**Frequency of data input by participant:** Once daily.<br><br>**Real-time feedback:** Unclear, 2-4 text messages per day.<br><br>**Additional software:** Web platform.<br><br>**Input from Clinician:** Intervention group: Mentors provided weekly telephone consultations over 6 weeks, average 15 minutes. The control group visited the hospital 3 times a week (1 hour education and 2x 1 hour exercise sessions).	
Waki et al (2014) [27]	57.25 years	41 male, 13 female.	9.05 years	No severe complications and had to be able to exercise. Potential participants' ability to use the system within 2 weeks and with training was assessed. 12 people were excluded because they could not use the system and devices properly. No comment on demographics of this group.	Participants in the DialBetics group received a smartphone (NEC, Tokyo, Japan: MEDIAS WP N-06C), NFC-enabled glucometer (Terumo, Tokyo, Japan: MS-FR201B) and Bluetooth-enabled BP monitor (Omron, Kyoto, Japan: HEM-7081-IT), pedometer (Omron HJ-720IT) with adapter (Omron HHX-IT1), and scale (Omron HBF-206IT), all<br><br>devices paired with a unique communicator that transmitted the readings by wireless network to the server. DialBetics is composed of 4 modules: 1. the data transmission module: patients' data—blood glucose, blood pressure, body weight, and pedometer counts—are measured at home and sent to the server twice a day right after the patients' measurement, the first 3 upon waking in the morning, then blood glucose, blood pressure, and pedometer readings at bed time. 2. the evaluation module: data are automatically evaluated following the Japan	3 month intervention, 3 month follow-up

Diabetes Society (JDS) guideline's targeted values—optimally, blood glucose below 110 mg/dl before breakfast, below 140 mg/dl at bed time; blood pressure below 130/80

mmHg; and pedometer count above 10,000. determines if each reading satisfies guideline requirements, then immediately sends those results to each patient's smartphone. 3. the communication module: (a) the patient's voice/text messages about meals—main dish of a meal—and exercise that is not counted by a pedometer—the type of exercise and its duration—are sent to the server. 4. Nutritional value calculation: photos of meals uploaded were reviewed by dieticians.

Frequency of data input by participant: Twice daily

Real-time feedback: Yes, automated messages based on data input.

Additional software: First known study to incorporate natural language processing. Patients were able to input some data (about meals) by text or by voice message and the system analysed the data.

Input from Clinician: Dieticians reviewed the photos of meals uploaded and sent patients messages around twice a week. Readings defined as abnormal—blood glucose above 400 mg/dl or below 40 mg/dl, and systolic blood pressure above 220 mmHg—are reported to a doctor as "Dr Call," meaning a physician will check the data and interact

with the patient if necessary.
